# Investigation on Surface Tilting in the Failure Process of Shallow Landslides

**DOI:** 10.3390/s20092662

**Published:** 2020-05-06

**Authors:** Shifan Qiao, Chaobo Feng, Pengkun Yu, Junkun Tan, Taro Uchimura, Lin Wang, Junfeng Tang, Quan Shen, Jiren Xie

**Affiliations:** 1 Department of Civil Engineering, Central South University, Changsha 410075, China; qiaosf@csu.edu.cn (S.Q.); fengchaobo@csu.edu.cn (C.F.); frankyupk@csu.edu.cn (P.Y.); 184801006@csu.edu.cn (J.T.); 2 Department of Civil and Environmental Engineering, Saitama University, 255 Shimo-Okubo, Sakura-ku, Saitama 338-8570, Japan; uchimura@civil.t.u-tokyo.ac.jp (T.U.); tang.j.360@ms.saitama-u.ac.jp (J.T.); 3 Chuo Kaihatsu Corporation, Nishiwaseda1-13-5, Shinjuku, Tokyo 169-8612, Japan; wang@ckcnet.co.jp; 4 Department of Civil Engineering, Hunan University of Technology, Zhuzhou 412000, China; shenquan123456@csu.edu.cn

**Keywords:** tilting sensor, slope failure, tilt direction, landslides, early warning systems

## Abstract

In recent decades, early warning systems to predict the occurrence of landslides using tilt sensors have been developed and employed in slope monitoring due to their low cost and simple installation. Although many studies have been carried out to validate the efficiency of these early warning systems, few studies have been carried out to investigate the tilting direction of tilt sensors at the slope surface, which have revealed controversial results in field monitoring. In this paper, the tilting direction and the pre-failure tilting behavior of slopes were studied by performing a series of model tests as well as two field tests. These tests were conducted under various testing conditions. Tilt sensors with different rod lengths were employed to investigate the mechanism of surface tilting. The test results show that the surface tilting measured by the tilt sensors with no rods and those with short rods located above the slip surface are consistent, while the tilting monitored by the tilt sensors with long rods implies an opposite rotational direction. These results are important references to understand the controversial surface tilting behavior in in situ landslide monitoring cases and imply the correlation between the depth of the slip surface of the slope and the surface tilting in in situ landslide monitoring cases, which can be used as the standard for tilt sensor installation in field monitoring.

## 1. Introduction

Landslides frequently occur around the world every year and pose a large threat to people’s lives and property. Typical methods for landslide prevention, such as retaining walls and ground anchors, have been employed to reduce the damage caused by landslides. However, these typical methods are costly and not suitable for a large number of slopes with potential risks of failure.

On the other hand, some early warning systems have been proposed based on the correlation between the rainfall intensity and risk of landslides [1,2,3,4]. These early warning systems work well in evaluating the likelihood of potential landslides in a region, but they are not effective in predicting a specific landslide within a region. Rainfall can change the soil moisture content, which can then affect the soil strength and trigger a landslide. Other early warning systems were also developed using the soil moisture index [4]. However, the relationship between the soil moisture content and stability of slopes is abstruse.

When a soil slope is deformed, energy is released in the form of elastic waves owing to the movement or fracturing of soil particles. Hence, some researchers focus on the application of acoustic emission (AE) technology in the monitoring of landslides to capture elastic waves [5,6,7,8,9,10,11]. However, the effectiveness of such systems to predict landslides is still under investigation. The installation of these systems is also complicated as a borehole and an active waveguide are needed.

Ground-based interferometric synthetic aperture radar (GB-InSAR) is a new tool that has been used to monitor landslide motion and can reduce losses caused by landslides by providing early warnings [12,13,14]. However, the cost of this type of monitoring system is also expensive for wide usage.

Typical monitoring systems of landslides monitor the displacement of soil from slopes [12,15,16,17,18,19,20,21] by utilizing conventional geotechnical monitoring methods, such as wire extensometers, borehole tilt meters or inclinometers. Some prediction methods for these types of early warning systems have also been proposed [22,23,24,25,26,27]. However, the inconvenience of installation and maintenance of these monitoring systems [28,29] limits their wide-scale application.

Reducing costs and price is still a primary concern in the application of early warning systems. The prices of some measuring equipment, such as extensometers, borehole tilt meters or devices to build acoustic emission monitoring systems, are still too high, which makes the price of the entire monitoring system unacceptable for wide-scale applications. Cable connections for such types of systems are also inconvenient for installation and difficult to maintain, which may also increase the cost.

In recent years, with the development of microelectronic techniques, early warning systems utilizing MEMS (microelectromechanical systems) technology have been proposed to monitor and estimate the risk of slope failure [28,29,30,31,32,33,34,35,36,37,38,39,40,41,42,43]. Compared to conventional instruments used to monitor landslides, MEMS sensors, such as accelerometer sensors [30,31,32] and tilt sensors [33,34,35,36,37,38,39], are small, inexpensive and sophisticated, which also greatly reduces the cost of building early warning systems. The low-power radio communication and modular design make the installation and maintenance of the whole system more convenient than cable-connected devices, and the data transmission is more efficient. De Dios et al. [33] and Marciano Jr [34] proposed a landslide monitoring system composed of a “sensor column” buried vertically underground in a borehole. The sensor column consists of pipe segments, each containing triaxial accelerometers for measuring tilt and capacitive sensors for water content measurements. Measurements of the internal deformation taken in each segment are accessed via a controller area network (CAN) communications protocol. However, the tilt meter in the borehole is not convenient to install owing to the requirement of a borehole.

Different systems based on monitoring the tilting behavior of the soil surface with inexpensive and sophisticated sensors have been proposed [28,29,30]. The tilt sensors in the monitoring systems proposed by Dikshit et al. [36] are buried under the soil surface. The tilt sensors in the monitoring systems for shallow landslides proposed by the authors [37,38,39] are attached to a rod insert into the unstable soil layer on the slope surface at a depth of approximately 1 m or more. Accordingly, the tilting behavior reveals the movement of the rod with the landslide mass, and the installation is much simpler than that of a tilt meter. A series of model tests and field tests were conducted. The results show a correlation between the abnormal behaviors in the tilting history on the slope surface and the slope failure [39,40,41,42,43]. However, some tilt sensors located above the slip surface of the landslides with rotational components tilted backward when the slope was sliding, while the tilt sensor together with a rod reaching the slip surface of slopes tilted forward during the failure process [42]. This phenomenon may be mainly affected by the length of the rod. If the rod is long enough to insert into the stable layer, the upper part of the rod will rotate with the landslide mass, which leads the tilt sensor to tilt forward. If the rod is short, the tilt sensor will rotate with the landslide mass. Although this behavior has been detected, there are few studies exploring this problem.

In this paper, to investigate the tilting behavior of tilt sensors with different rod lengths as landslides develop, a series of model tests were conducted, and different experimental parameters were considered. In addition, two field tests were also carried out, in which slope failure was triggered by artificial rainfall. In all of these tests, the tilting behaviors of the slopes along the slope direction were monitored by tilt sensors connected to a data logger for continuous data recording. The frequency for data sampling in the model tests and the field tests was 1/60 Hz.

## 2. Proposed Equipment for Slope Monitoring

**Figure 1:**
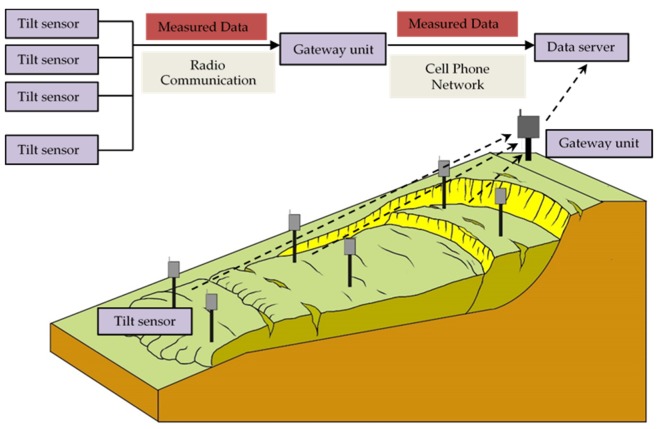
Outline of the wireless monitoring and early warning system for slope failures.

The early warning system proposed by the authors consists of a group of sensor units and a gateway unit for a slope region, as shown in Figure 1. The sensor units are installed at shallow depths on the slope, and these units are designed to be wireless. The sensor units periodically measure the condition of the slope at 10 min intervals. The collected data are transferred from the tilt sensors to the gateway unit by radio communication, and then are sent to a data server on the internet through a cellular network. Then, the data can be browsed on the website and will also be processed on the server. If an abnormal behavior in the slope is detected, a warning will be issued.

**Figure 2:**
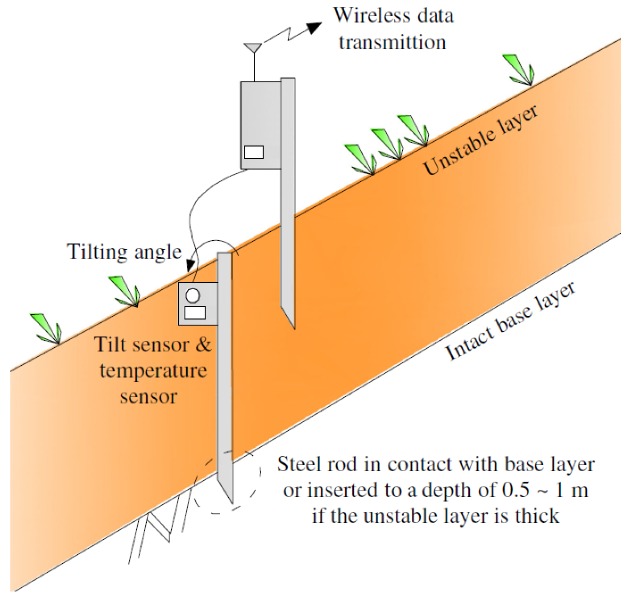
Early warning system using slope tilting.

As shown in Figure 2, the surface tilt sensor developed by the authors is equipped with a MEMS tilt sensor (nominal resolution = 0.0025° = 0.04 mm/m). An additional temperature sensor is also used for temperature compensation in the tile sensor. Each sensor unit is powered by 4 AA alkaline batteries and functions well in the field for a duration of more than one year. By connecting an optional solar battery, which costs approximately 5 USD, the sensor unit can work semi-indefinitely. The tilt sensor is attached to a rod vertically installed in the slope surface.

## 3. Methodology

To investigate the tilting behavior of tilt sensors under the conditions of different experimental parameters, the current investigations involved ten small-scale model tests using tilt sensors without rods, five small-scale tests model using tilt sensors with short rods, two small-scale model tests using tilt sensors with long rods and two field tests.

### 3.1. Laboratory Model Tests

**Figure 3:**
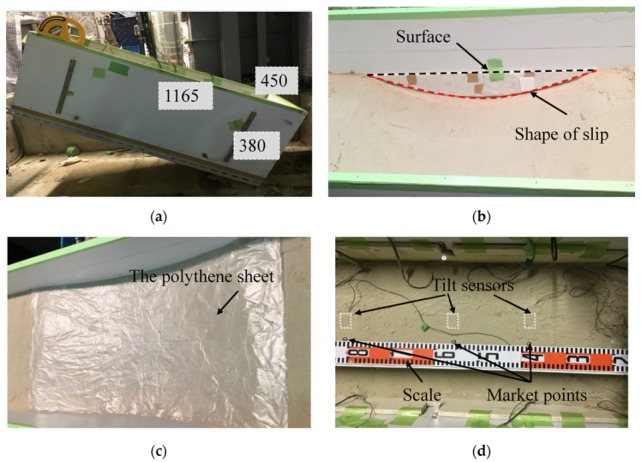
Images of the laboratory test steps. (**a**) The dimensions of the container; (**b**) the creation of the slip surface; (**c**) the polyethylene sheet; and (**d**) the built surface layer and the installed instruments.

**Figure 4:**
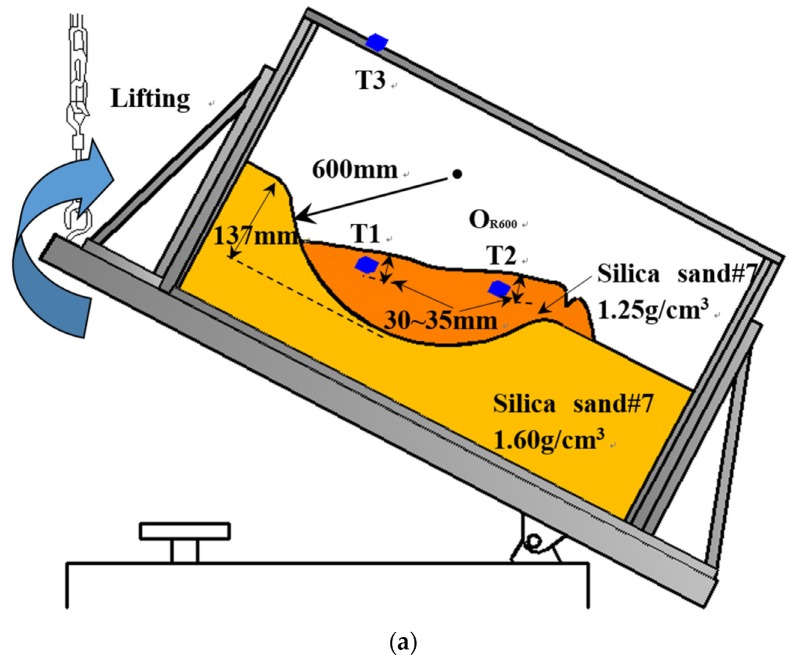

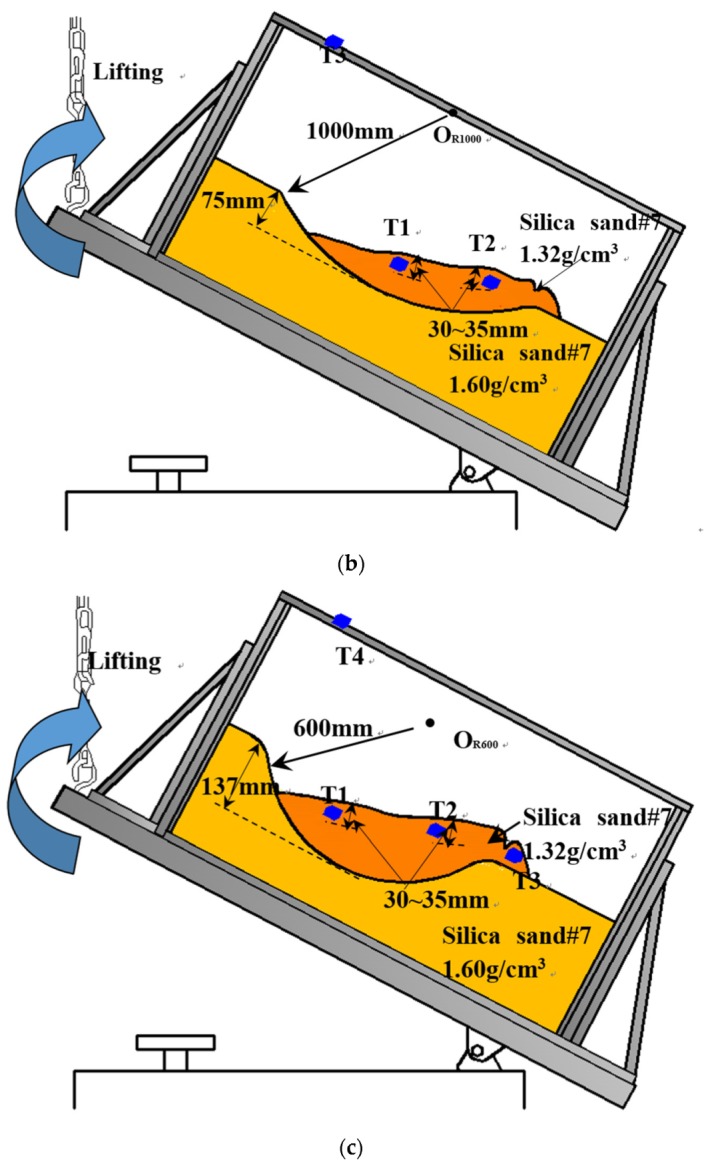

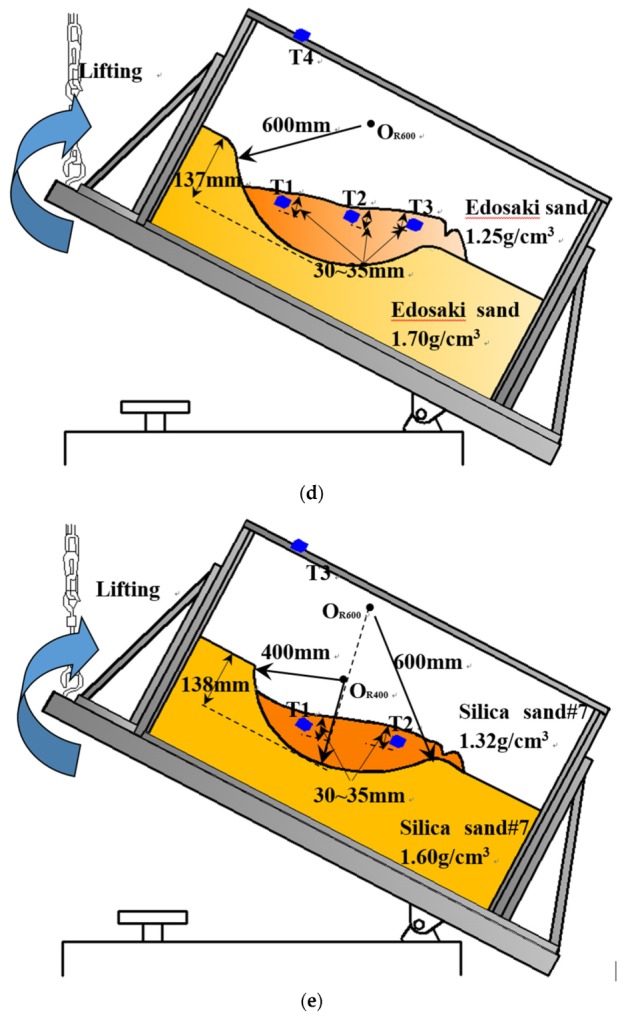

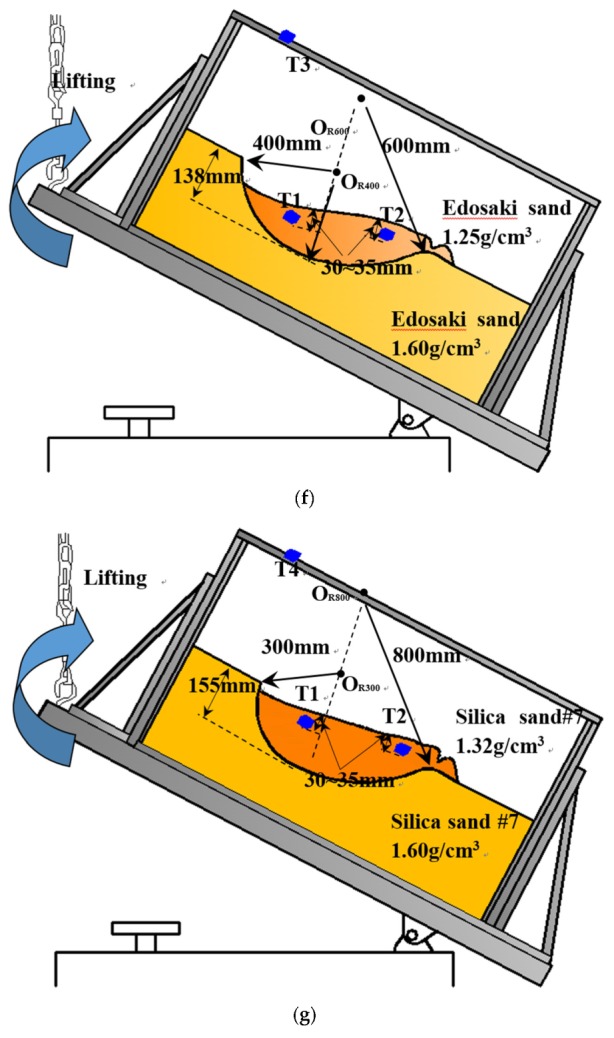

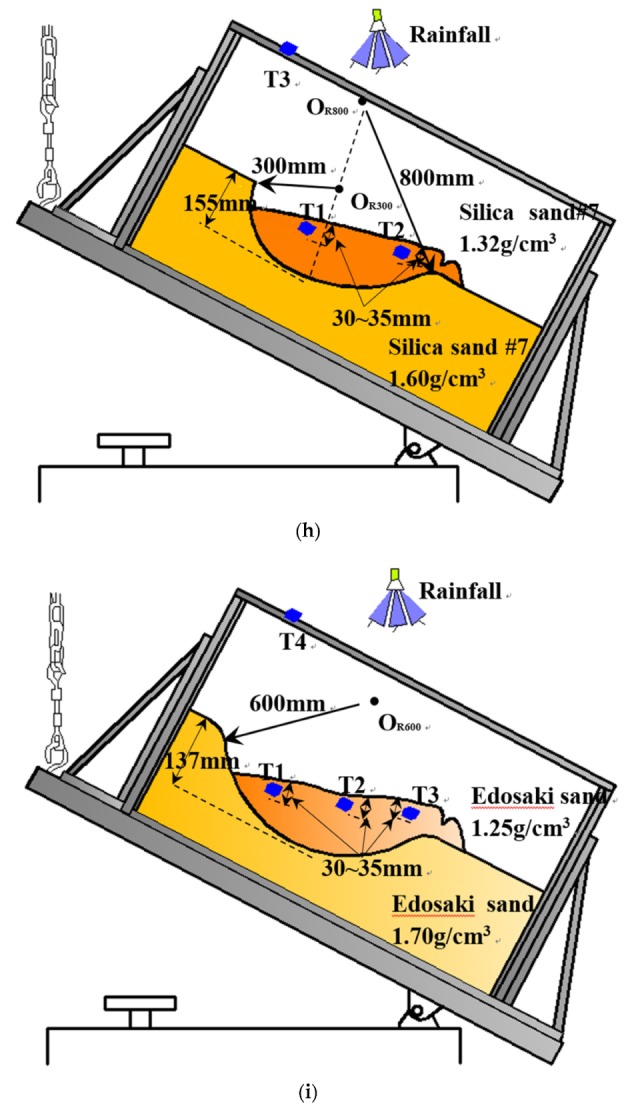

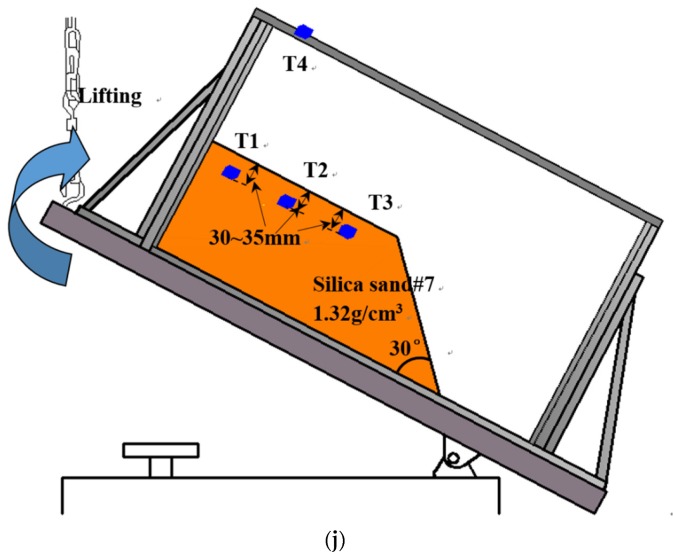
The cross sections of the slope models and the setups of the apparatuses in Test 1 to Test 10 as shown from (**a**) to (**j**).

**Figure 5:**
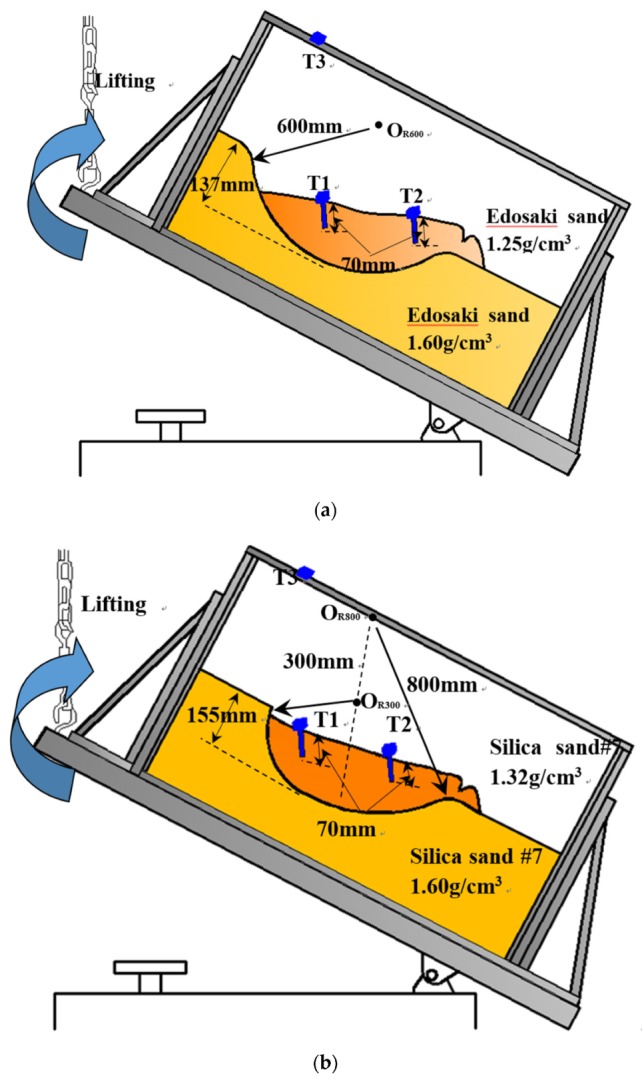

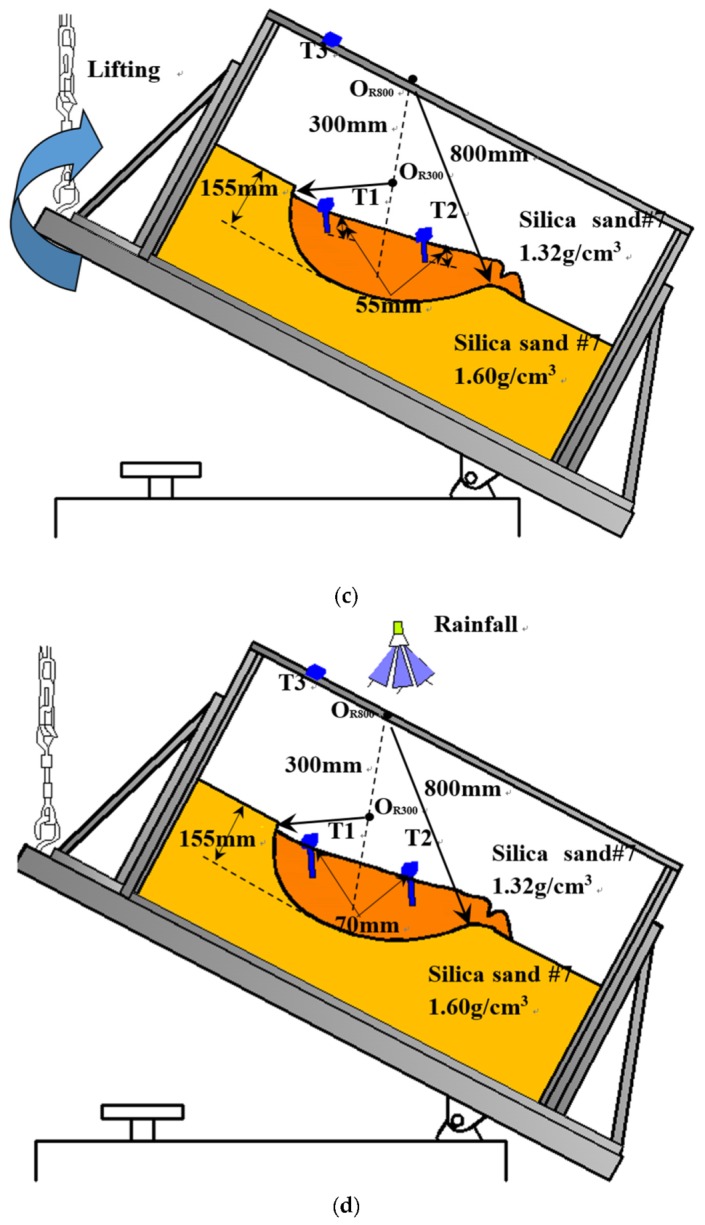

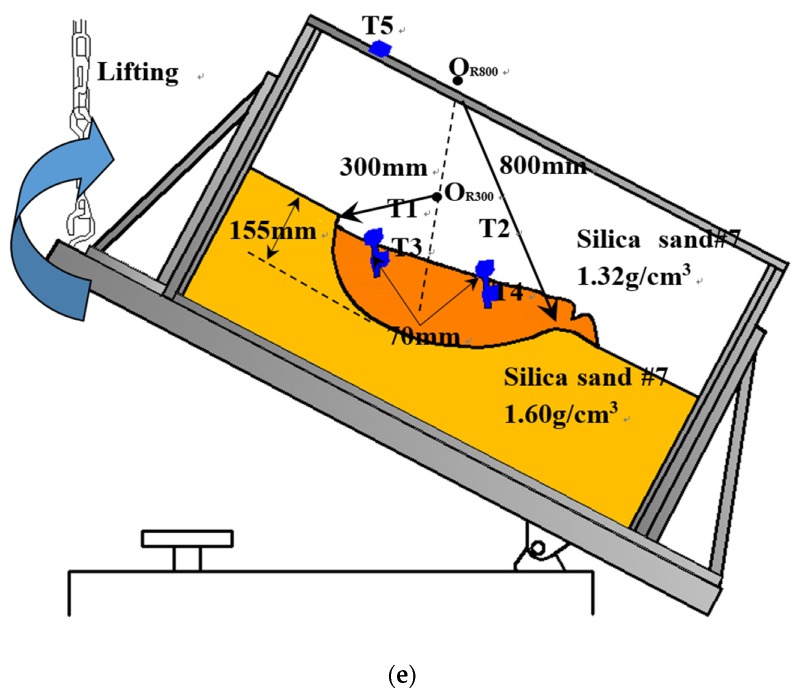
The cross sections of the slope models and the setups of the apparatuses in Test 11 to Test 15 as shown from (**a**) to (**e**).

**Figure 6:**
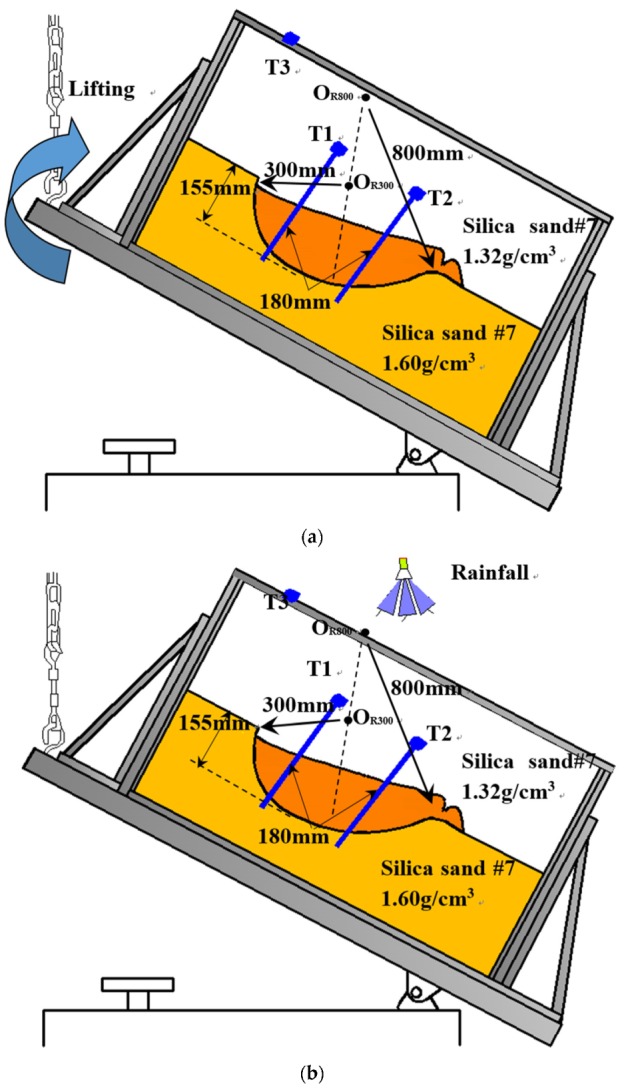
Illustration of using tilt sensors with long rods reaching the slip surface in (**a**) Test 16 and (**b**) Test 17.

The slope model in these tests was built in a rectangular box, measuring 1165 mm (length) × 450 mm (width) × 380 mm (height).The soil was divided into two parts: the base layer and the surface layer. The slope construction procedure are presented in Figure 3. First, to make the base layer, the sand used in the model tests was compacted to a certain density by tamping. Then, the base layer was carved into a predesigned shape. The predefined shape was curvilinear and consisted of a semicircle parts or two circular parts with specified radii. After this step, the surface layer was built using the same sand used to build the base layer with the specified density. A polyethylene sheet was placed between the base layer and surface layer and acted as the predefined slip surface of the slope models before the surface layer was built, as polyethylene can reduce friction and restrict water flow. After building the slope model, tilt sensors were installed in the designated position, which are illustrated in Figure 4, Figure 5 and Figure 6. In addition, an extra tilt sensor was fixed on the wooden box to measure the tilting angle of the box. In addition, in contrast to other model tests, a planar slip surface was built to investigate the tilting behaviors in translational landslides in test 10. Considering that the complex curve of landslides can be decomposed into different arcs with different radii for simplification, the slip surfaces in the model tests are assumed to be semicircular or are assumed to have two quadrants with different radii.

Two landslide-triggering factors were considered in the model tests: artificial rainfall and inclining the slope by lifting one end of the wooden container step-by-step. At the same time, an external tilt sensor was attached to the container to measure the tilting angle of the box to calibrate the tilt angle of the sensor when the wooden container was inclined and to record the initial angle of the container when using the artificial rainfall method to trigger a landslide. As the trigger factor is active, the tilting behaviors were obtained by recording the data transferred from the tilt sensors.

All of the details of the model test mentioned above are shown in Figure 3, and the type of sands and their relative densities, the radii of the predefined slip surfaces and the landslide-triggering factors designed for each model test are introduced in detail in Table 1, Table 2 and Table 3.

#### 3.1.1. Small-Scaled Model Tests Using Tilt Sensors without Rods

The specific testing conditions, including the geometries of the predefined slip surfaces, the materials used in the model tests and their densities and landslide-triggering factors, are listed in Table 1. The schematic illustration of the cross sections of the slope models and the arrangements of the apparatuses employed in these tests are presented in Figure 4. As shown in Figure 4, tilt sensors were installed on the slope, and an external tilt sensor was attached to the container. As illustrated in Table 1, in Tests 1–7 and Test 10, slope sliding was triggered by inclining the wooden container step-by-step, while artificial rainfall was applied in Test 8 and Test 9 to induce sliding.

#### 3.1.2. Small-Scaled Model Tests Using Tilt Sensors with Short Rods

Model tests using tilt sensors with short rods installed above the predefined slip surface were conducted to investigate the tilting direction of the tilt sensors installed on slopes. The details about the geometries of the predefined slip surfaces, the materials used in the model tests and their densities and landslide-triggering factors are listed in Table 2. The schematic illustration for the cross sections of the slope models and the arrangements of the apparatuses employed in these tests are presented in Figure 5.

#### 3.1.3. Small-Scaled Model Tests Using Tilt Sensors with Long Rods Reaching the Slip Surface

Two model tests were designed to investigate the tilting behaviors of sensors with long rods when landslides are triggered by changes in the stress field through lifting the model test box and changes in the water content induced by simulated rainfall. The geometry of the predefined slip surface and details of the materials used in these tests, as well as the landslide-triggering factors, are listed in Table 3. The schematic illustration for the cross sections of the slope models and the arrangements of the apparatuses employed in these tests are presented in Figure 6.

### 3.2. Field Tests

**Figure 7:**
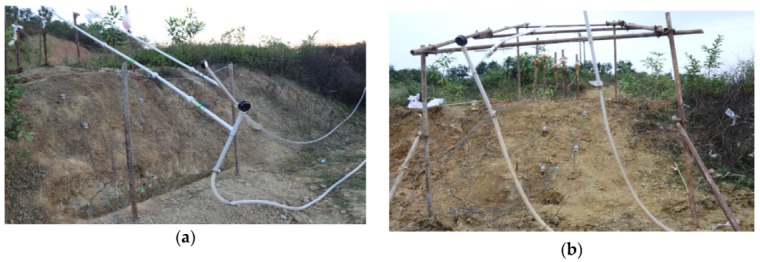
Images for (**a**) Field Test 1 and (**b**) Field Test 2.

**Figure 8:**
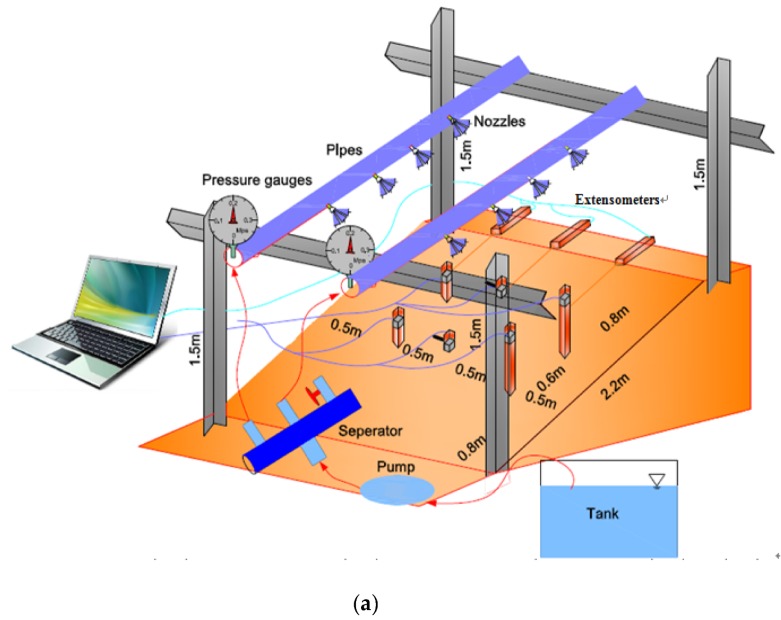

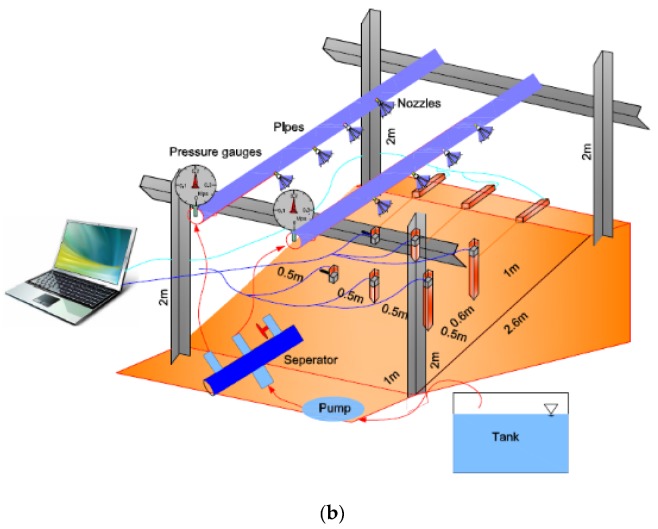
Illustration of the arrangement of the sensors in (**a**) Field Test 1 and (**b**) Field Test 2.

The site for the field tests, which consists of weak expansive soil and some plant roots, is located in Guangxi Province, and the slope angle is 43 degrees. Trenches were excavated both at the toe and the crest of this slope to a depth of 0.4 m. Rainfall was supplied by an artificial rainfall supply system consisting of a pump, artificial pond, water separator and nozzles. In Field Test 1, the rainfall intensity was set to 40 mm/min. Tilt sensors with different lengths of rods, 50 mm, 300 mm and 800 mm, were installed on the slope. The arrangement of the sensors and geometries of the slopes are shown in Figure 7a and Figure 8a. The rainfall intensity in Field Test 2 was 21 mm/h, lower than that in Field Test 1. There were two types of rods with different lengths in Field Test 2, 30 mm and 300 mm. The arrangement of the sensors and geometry of the slope are different in Field Test 2, as shown in Figure 7b and Figure 8b.

## 4. Results and Discussion

### 4.1. Model Tests

#### 4.1.1. Small-Scale Model Tests Using Tilt Sensors without Rods

**Figure 9:**
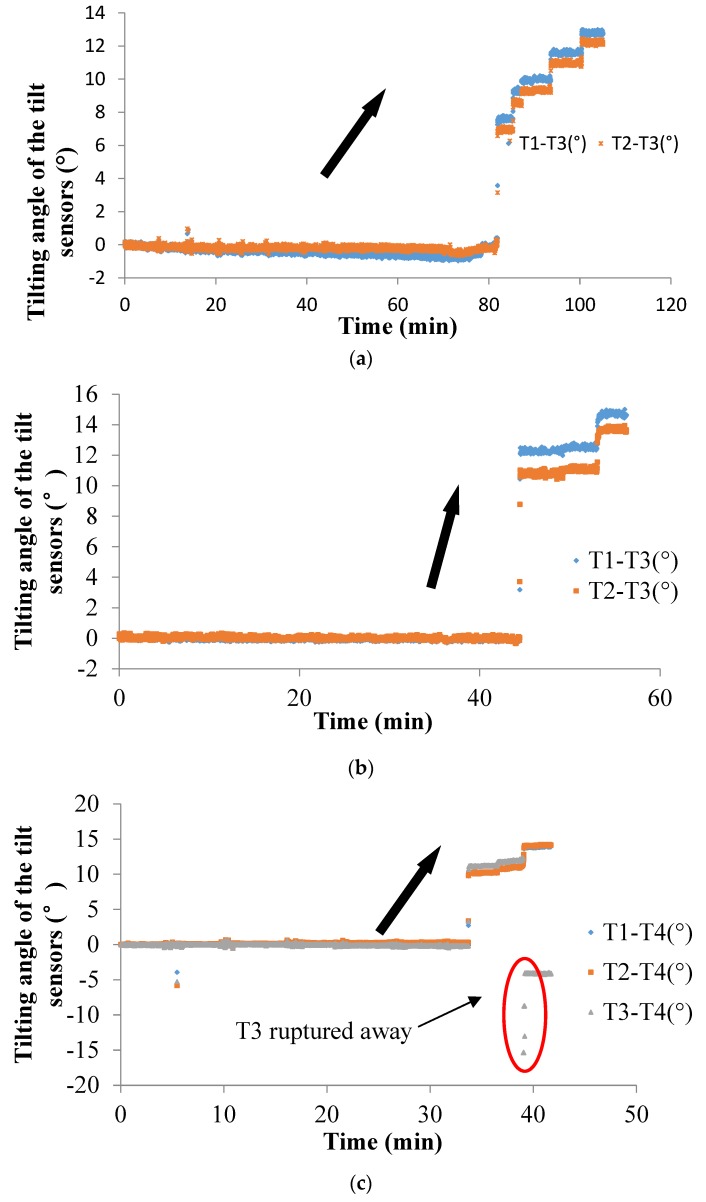

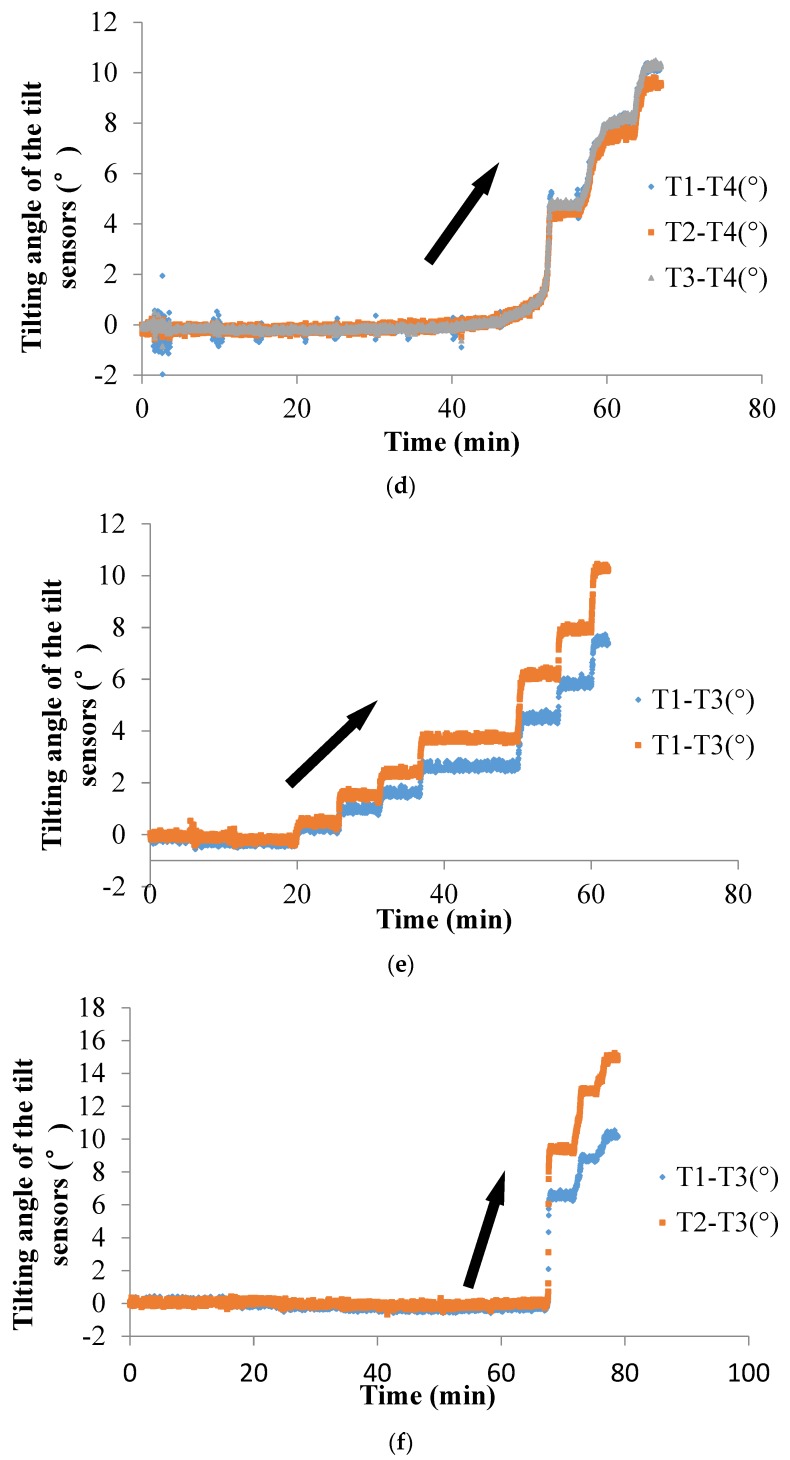

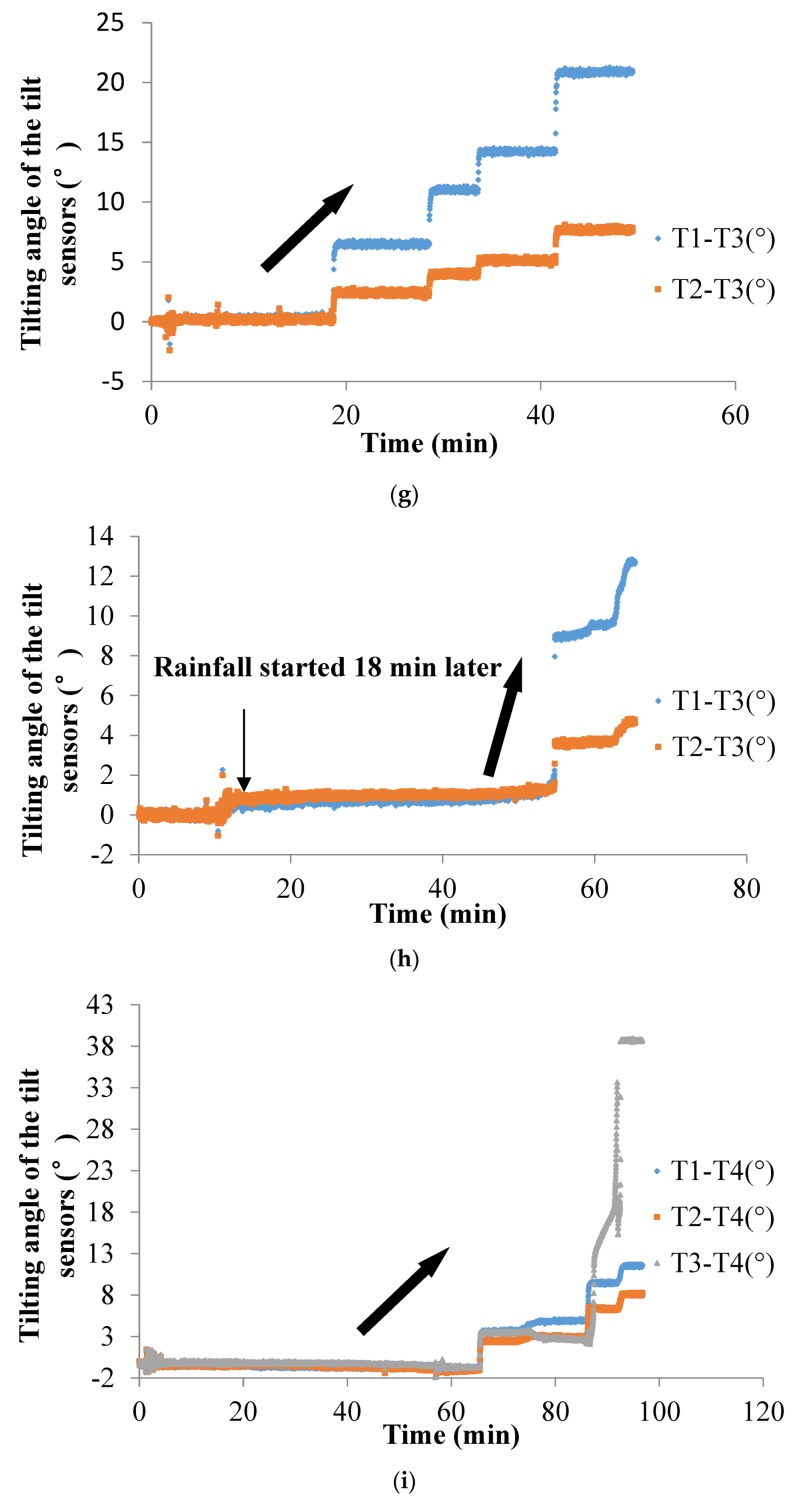

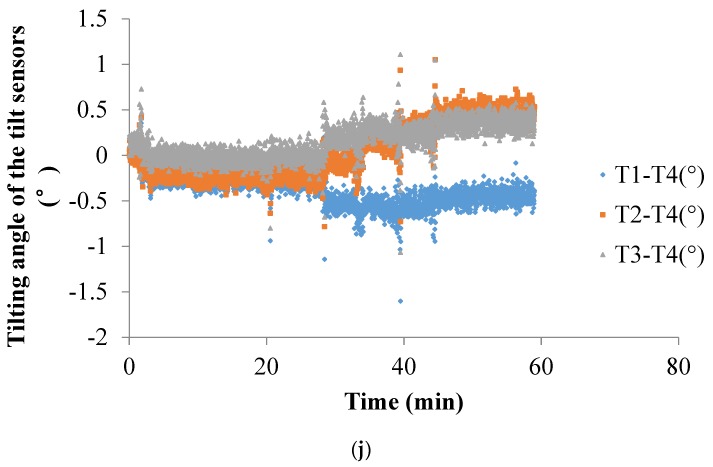
Temporal history of the slope-tilting degree recorded in Test 1 to Test 10 as shown from (**a**) to (**j**).

The landslides in Tests 1−8 were caused by inclining the wooden container step-by-step, and the landslip in Tests 9 and 10 was caused by artificial rainfall. The difference between the data obtained from the tilt sensor installed in the surface layer and that from the external tilt sensor attached to the edge of the container represents the tilting angle induced by slope sliding, as shown in Figure 9. As illustrated in Figure 9, the tilt sensors without rods show the same rotation trend as the landslide mass regardless of the varying test conditions. To a certain extent, the behavior of the tilt sensor without rods represents the behavior of the soil in a landslide mass because the tilt sensors are embedded in the soil and small enough to ignore their effect on the soil properties. However, there exists an exception in Test 3, as shown in Figure 9c. The tilt sensor marked as T3 shows an abnormal tilting behavior compared to the other tilt sensors because of the slope rupture at the bottom part of the slope model caused the uncertainty behavior of the tilt sensor, as shown in Figure 4c. Thus, the data from the tilt sensor T3 dropped suddenly. As illustrated in the data collected, the tilting angle of the sensors shows an abrupt increasing trend as the landslide developed, and the position of the tilt sensors and the landslide-triggering factors do not affect the trend in the change in tilting angle obtained by the tilt sensors.

In Test 10, the changing tilting angle of the sensor is not obvious in the translational landslide, but the tilt sensor near the edge of the slope shows a different rotating direction compared to the other two tilt sensors. This phenomenon is caused by the complex behavior of the soil when the slope collapses, especially when the soil is near the edge of the slope. Thus, the tilting behavior of the tilt sensor of a translational landslide is uncertain.

#### 4.1.2. Small-Scale Model Tests Using Tilt Sensors with Short Rods

**Figure 10:**
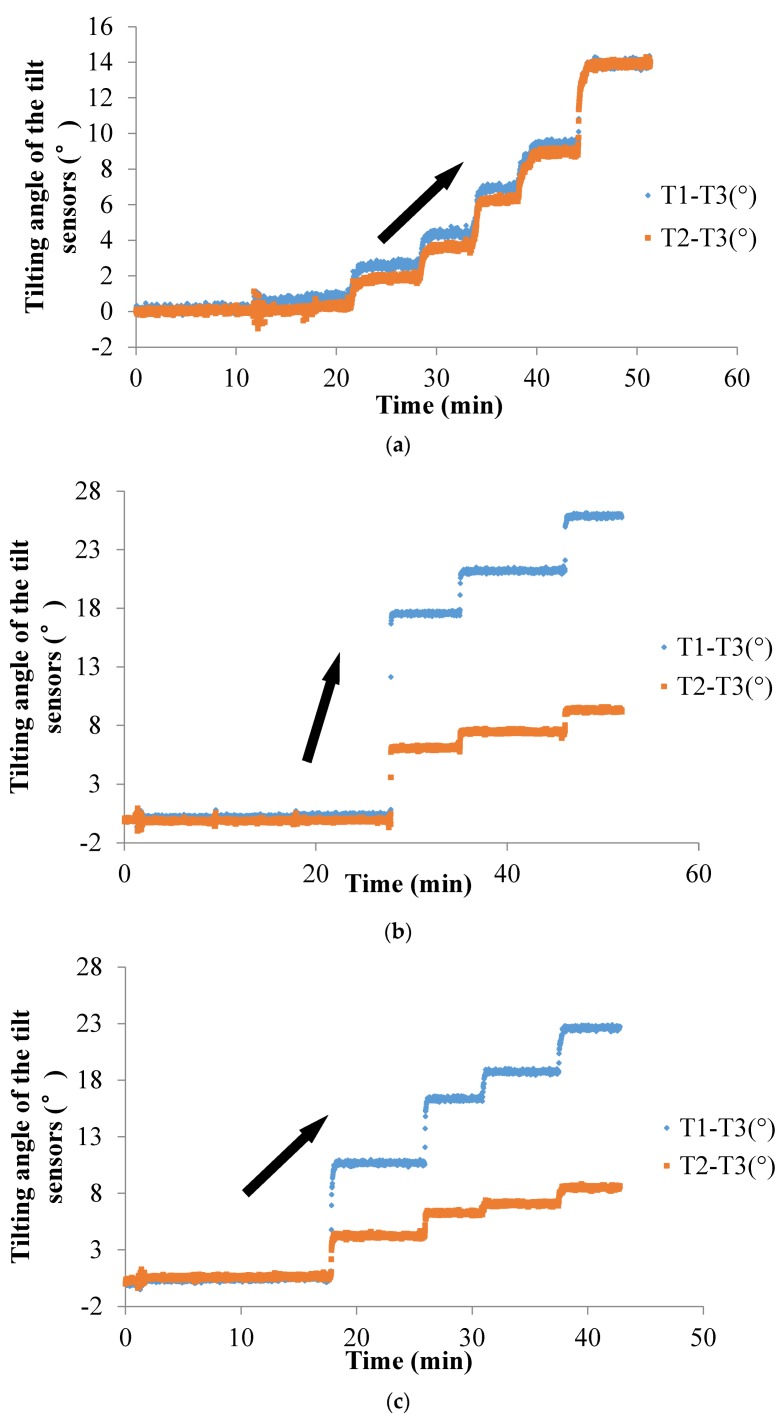

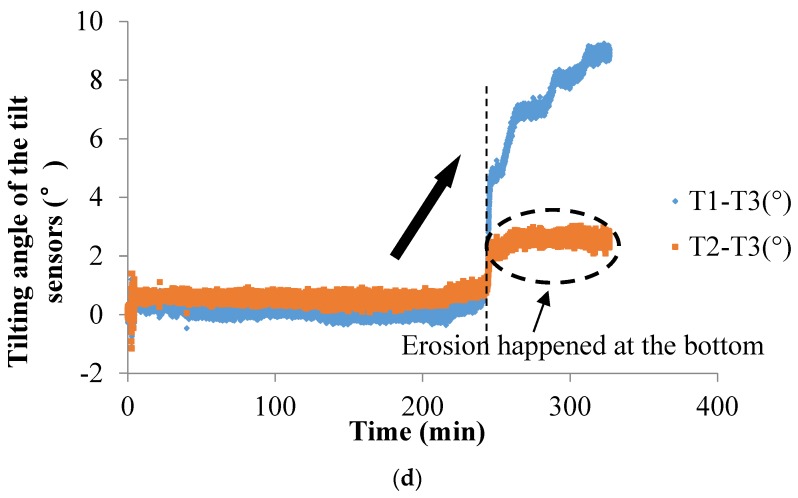
Temporal history of the slope-tilting degree recorded in Test 14. Test 11 to Test 14 as shown from (**a**) to (**d**).

The data of the temporal history of the tilting angle obtained by the tilt sensors in Tests 11–15 are plotted in Figure 10. As Figure 10 illustrates, the tilt sensors with the short rods show the same rotation trend as the landslide masses, which was not affected by the trigger factor, radius of the slip surface, relative density or type of soil. This tilting behavior of the tilt sensors with the short rods is the same as that of the tilt sensors without the rods mentioned above, and the tilting angle of the sensors also shows an abrupt increasing trend as the landslide developed. The position of the tilt sensors and the landslide-triggering factors also do not affect the trend in the change in the tilting angle obtained by the tilt sensors, as shown in the temporal history of the slope-tilting experiments.

**Figure 11:**
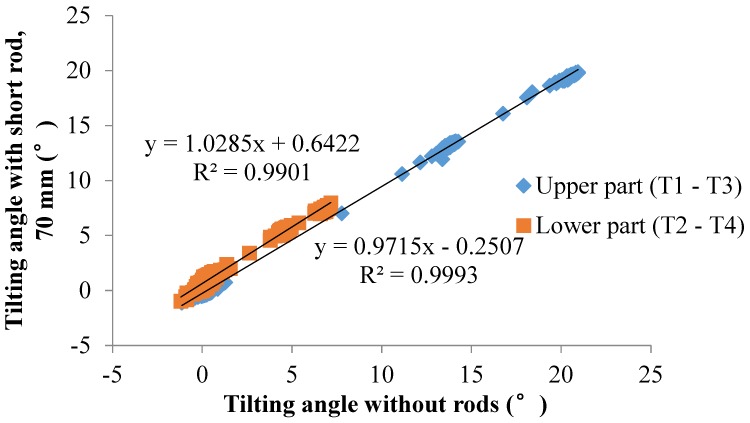
The relationship between the tilt angle measured by sensors without rods and sensors with short rods in Test 15.

Five tilt sensors were used in Test 15. The tilt sensor marked as T5 was attached to the wooden container to measure its tilting angle. The tilt sensors marked as T1 and T2 with 70 mm short rods were installed in the top part and bottom part of the slope. Additionally, the tilt sensors marked as T3 and T4 without rods were close to the locations of T1 and T2, respectively, as illustrated in Figure 5f. Based on the regression analysis, there is an approximate linear relationship between the tilt angle of the tilt sensors with short rods (T1 and T2) and that of the tilt sensors without rods (T3 and T4), as shown in Figure 11. The relationship between T1 and T3 can be expressed as y = 0.9715x − 0.2507, and the relationship between T1 and T3 can be expressed as y = 1.0285x − 0.6422, as plotted in Figure 6. In these two equations, x denotes the tilt angle of the tilt sensor with a short rod (T1 or T2), and y denotes the tilt angle of the tilt sensor without a rod (T3 or T4). Tilt sensors with no rods or with short rods installed above the slip surface show a similar tilting behavior during slope sliding, as the two equations express, which means that the tilt sensors with short rods can reveal the tilt behavior of soil in a landslide mass, and that the tilt sensors do not have to be embedded in the soil but can be attached to a rod that sticks into the soil as a more convenient way to install the tilt sensors.

#### 4.1.3. Small-Scale Model Tests Using Tilt Sensors with Long Rods Reaching the Slip Surface

**Figure 12:**
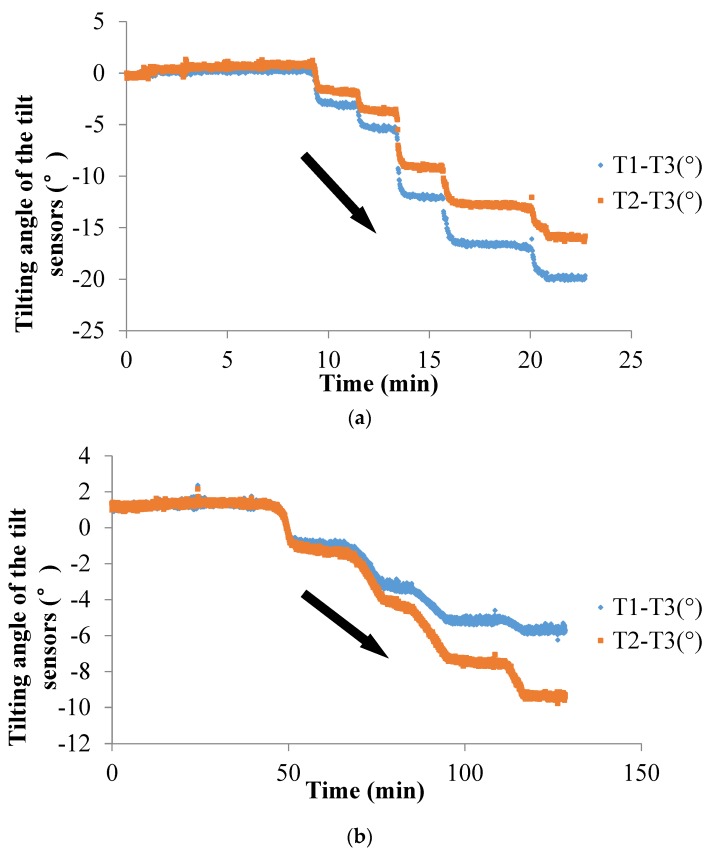
Temporal history of the slope-tilting degree recorded in (**a**) Test 16 and (**b**) Test 17.

The results of the temporal history of the tilting angle plotted in Figure 12 show that, regardless of the landslide-triggering factor, the sensors with long rods have an opposite rotational trend with the landslide masses when landslides occur. Because part of the rod is in the sliding mass, the lower part of the rod is embedded in the soil that does not move, and the upper part of the rod moves forward with the landslide mass, which leads to the rod tilting forward, which was observed in these two small-scale model tests. This behavior is also observed by Xie et al. [42], who found that the tilt sensor located above the slip surface of the landslide with rotational components tilted backward when the slope was sliding, while the tilt sensor together with a rod reaching the slip surface of slopes tilted forward in the failure process. In this study, the results show that the tilt direction of the rods is not affected by the landslide-triggering factors and other soil parameters considered in the model test but is determined by the position of the rod related to the slip surface. In addition, the tilting angle of the sensors also shows an abrupt increasing trend as the landslide develops. Moreover, the position of the tilt sensors and the landslide-triggering factors do not affect the trend in the change in the tilting angle. However, the tilt directions of the tilt sensors are different than those of the tilt sensors without rods or with short rods.

### 4.2. Field Tests

**Figure 13:**
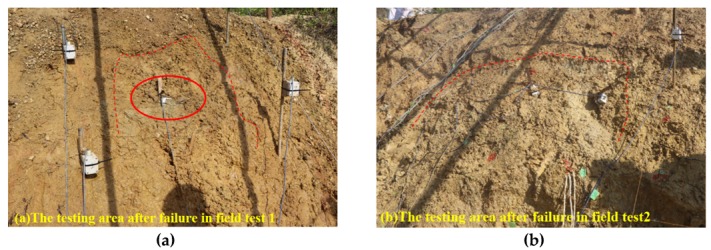
Images of the testing area before and after slope failure: (**a**) the testing area after failure in Field Test 1; (**b**) the testing area after failure in Field Test 2.

**Figure 14:**
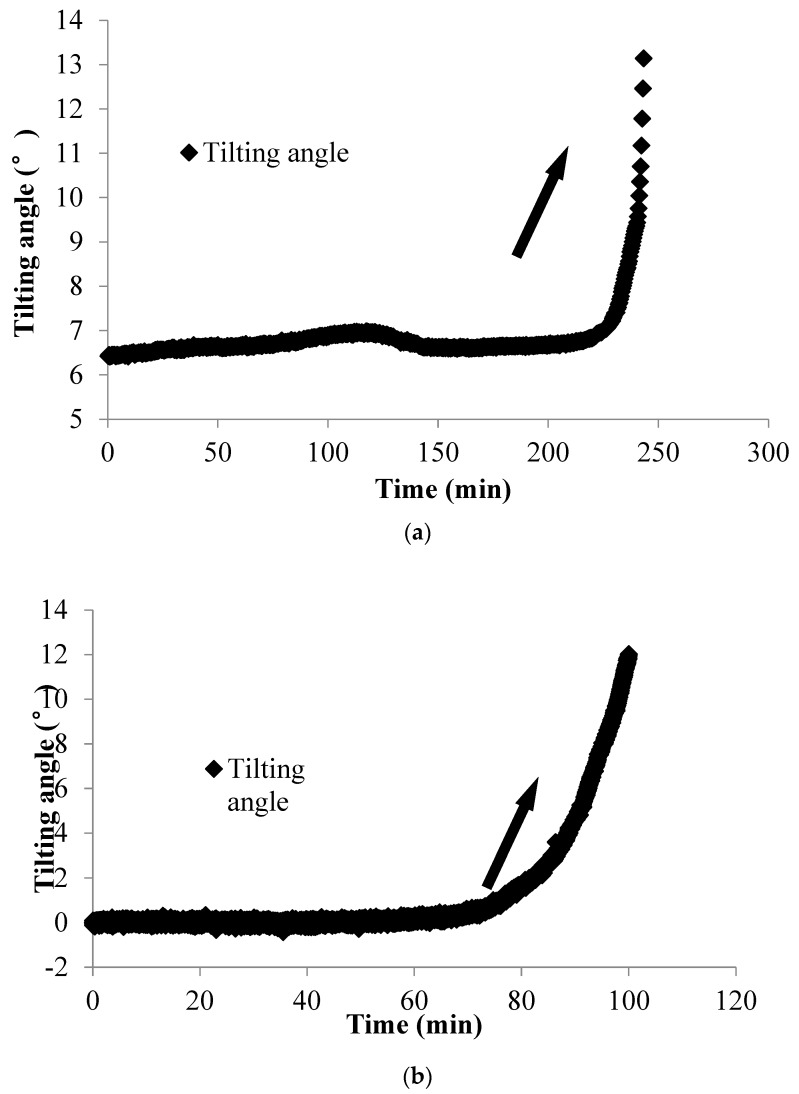
Temporal history of the slope-tilting degree recorded by a tilt sensor with a rod 50 mm long located in the failed area in (**a**) Field Test 1 and (**b**) Field Test 2.

**Figure 15:**
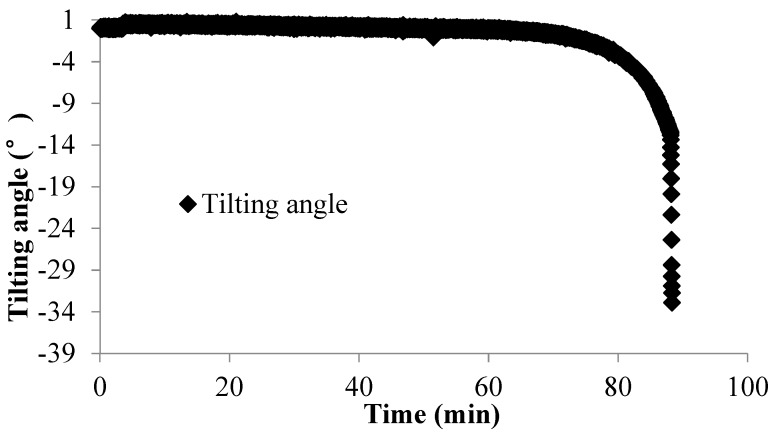
Temporal history of the slope-tilting degree recorded by the tilt sensor with a rod 300 mm long located in the failed area of the slope in Field Test 2.

In Field Test 1, major deformation occurs in the top part of the slope with a steep scarp angle, as shown in Figure 13a. The depth of the slip surface of this slope is approximately 250 mm, which is deeper than the location of the failed tilt sensor. Consequently, the tilt sensor located in the failed area tilted backward, as presented in Figure 14a. This result is consistent with that of the model tests. In Field Test 2, the failure of the tilt sensors with rods of different lengths, 50 mm and 300 mm, installed in the lower part of the slope was caused by erosion, as shown in Figure 13b. Major deformation occurs in the top part of the slope, and the depth of the slip surface is also approximately 250 mm. As a result, the tilt sensor attached to a short rod, approximately 50 mm, tilted backward, as presented in Figure 14b, while the tilt sensor attached to a longer rod, approximately 300 mm, tilted forward, as shown in Figure 15; the length of the long rod is greater than 250 mm, the depth of the slip surface. This result is also consistent with the analysis of the results obtained from the model tests. The tilting angle of the sensors also shows an abrupt increasing trend as landslides develop, which can also be observed from the model test. In general, these test results, obtained from the model and field tests, can reveal that the abnormal tilting in the slope surface is a precursor of slope failure.

### 4.3. Discussion

Surface tilting was tentatively considered an implication for the prediction of landslides with rotational components [26,44]. From the authors’ previous study, the tilting rate also shows a sharp increase at a shorter duration before slope failure [39,40,41,42,43]. In this study, a sharp increase in the tilting rate of tilt sensors before landslides is also observed, as shown in Figure 9, Figure 10, Figure 12, Figure 14 and Figure 15. Therefore, this study suggests that tilt behavior is an effective index to predict and monitor landslides.

Longstanding efforts have been made to predict the slip surface of slopes, and some methods have been proposed in recent decades based on surface displacements and morphology [45,46,47,48,49]. The computational process of those methods is complicated and involves many assumptions [49]. The authors’ previous study [43] revealed that the surface deformation due to the landslide masses is mainly caused by the slope rotation against the centers of the slip surfaces when the slope slides, and the path of the sliding masses is parallel to the slip surfaces. A method has also been proposed for the prediction of the slip surface, but this method does not consider the occurrence of different tilting directions by the tilt sensors, which has been observed but not previously investigated [42].

Based on the results of the model tests and field tests in this study, the tilting direction of the tilt sensors with long rods shows an opposite rotational trend with the landslide mass because if the lower part of the rod inserts into the stable part of the soil below the slip surface, the part of the rod in the landslide mass moves forward with the soil when a landslide occurs. Hence, tilt sensors with short rods may also show an opposite rotational trend with the landslide mass if part of the rod is inserted into the stable part of the soil below the slip surface. Thus, the depth of installation of the tilt sensors with rods should be wisely chosen for different types of landslides. For landslides with curved slip surfaces, both tilt sensors with short rods and tilt sensors with long rods can be used. For shallow translational landslides, tilt sensors with short rods are not effective. The rod of the tilt sensor needs to be inserted into the stable layer for monitoring these types of landslides, but further investigation is needed.

### 4.4. Limitations and Future Scope

Considering that a complex curve can decompose into different arcs with different radii for simplification, the slip surface in the model tests is assumed to be semicircular or is assumed to have two quadrants with different radii. However, in reality, the slide beds of the landslides also have types of lines and complex curves. The data in Test 10 of this study show that the tilt sensors are not suitable for monitoring translational landslides.

Hence, the proposed prediction method still has some drawbacks and limitations. First, further studies are needed to investigate the landslides of complex slide beds. Secondly, a detailed comparison with other technologies was not considered here and needs further investigation. Thirdly, this early warning system should be improved for monitoring translational landslides, and further studies are needed.

## 5. Conclusions

In this paper, the tilting direction of the tilt sensors attached to rods of different lengths installed on the surface of slopes was investigated by performing a series of model tests and field tests. The effects of different parameters on the tilting direction of the sensors were also investigated. The results revealed that the tilting angle of the tilt sensors with no rods and that of the tilt sensors with short rods show an approximate linear relationship during slope sliding. The tilting rate tended to increase, and a shorter duration remained before failure. This finding suggests that the tilt behavior is an effective index to predict and monitor landslides. However, the tilting direction of the tilt sensors with long rods shows an opposite rotational trend with the landslide mass because if the lower part of the rod is inserted into the stable part of the soil below the slip surface, the part of the rod in the landslide mass moves forward with the soil when a landslide occurs. The tilt sensors with long rods show a forward movement trend.

Hence, a tilt sensor with a long rod can be applied to monitor shallow translational landslides, but the end of the rod must be inserted into the stable layer; this still needs further investigation. A tilt sensor without rods and a tilt sensor with short rods can be applied to monitor shallow and rotational landslides. Both tilt sensors with short rods and tilt sensors with long rods can be used in landslides with curved slip surfaces, but tilt sensors with short rods are not effective in translational landslides.

In general, the results and previous studies prove that the early warning system the authors have proposed, or similar systems, can be effective, inexpensive and convenient to install to mitigate the losses caused by landslides. However, further studies and investigations are needed to perfect these types of systems, because the mechanism behind the sensors with rods rotating with the landslide mass is still unclear, and more data are needed to make the system adapt to complex situations that may occur in applications.

## Figures and Tables

**Table 1:** Small-scaled tests using tilt sensors without rods.

Test No.	Material	Radius of the Slip Surface R or R1 + R2 (mm)	Triggering Factor	Base Layer Density (g/cm^3^)	Surface Layer Density (g/cm^3^)	Depth (mm)
1	Silica sand #7	R: 600	Lifting	1.60	1.25	137
2	Silica sand #7	R: 1000	Lifting	1.60	1.32	75
3	Silica sand #7	R: 600	Lifting	1.60	1.32	137
4	Edosaki sand	R: 600	Lifting	1.70	1.25	137
5	Silica sand #7	R1 + R2: 600 + 400	Lifting	1.60	1.32	138
6	Edosaki sand	R1 + R2: 600 + 400	Lifting	1.60	1.25	138
7	Silica sand #7	R1 + R2: 300 + 800	Lifting	1.60	1.32	155
8	Silica sand #7	R1 + R2: 300 + 800	Rainfall	1.60	1.32	155
9	Edosaki sand	R: 600	Rainfall	1.70	1.25	137
10	Silica sand #7	Infinite (planar)	Lifting	/	1.32	/

**Table 2:** Small-scaled tests using tilt sensors with short rods

Test No.	Material	Radius of the Slip Surface (mm)	Triggering Factor	Base Layer Density (g/cm^3^)	Surface Layer Density (g/cm^3^)	Depth (mm)	Rod Length (mm)
11	Edosaki sand	R: 600	Lifting	1.60	1.25	137	70
12	Silica sand #7	R1 + R2: 300 + 800	Lifting	1.60	1.32	155	70
13	Silica sand #7	R1 + R2: 300 + 800	Lifting	1.60	1.32	155	55
14	Silica sand #7	R1 + R2: 300 + 800	Rainfall	1.60	1.32	155	70
15	Silica sand #7	R1 + R2: 300 + 800	Lifting	1.60	1.32	155	70 and 0

**Table 3:** Small-scaled tests using tilt sensors with long rods.

Test No.	Material	Radius of the Slip Surface (mm)	Triggering Factor	Base Layer Density (g/cm^3^)	Surface Layer Density (g/cm^3^)	Depth (mm)	Rod Lengths (mm)
16	Silica sand #7	R1 + R2: 300 + 800	Lifting	1.60	1.32	155	180
17	Silica sand #7	R1 + R2: 300 + 800	Rainfall	1.60	1.32	155	180
